# Differences in [^18^F]FDG uptake in BAT of UCP1 −/− and UCP1 +/+ during adrenergic stimulation of non-shivering thermogenesis

**DOI:** 10.1186/s13550-020-00726-x

**Published:** 2020-11-07

**Authors:** Christian T. McHugh, John Garside, Jared Barkes, Jonathan Frank, Constance Dragicevich, Hong Yuan, Rosa T. Branca

**Affiliations:** 1 Department of Physics and Astronomy, The University of North Carolina at Chapel Hill, Chapel Hill, NC USA; 2 Biomedical Research Imaging Center, The University of North Carolina at Chapel Hill, Chapel Hill, NC USA; 3 Department of Radiology, The University of North Carolina at Chapel Hill, Chapel Hill, NC USA

**Keywords:** Brown adipose tissue, Uncoupling protein 1, [^18^F]FDG, Thermogenesis, Standardized uptake value, Infrared thermography

## Abstract

**Background:**

Brown adipose tissue (BAT) is a fat tissue found in most mammals that helps regulate energy balance and core body temperature through a sympathetic process known as non-shivering thermogenesis. BAT activity is commonly detected and quantified in [^18^F]FDG positron emission tomography/computed tomography (PET/CT) scans, and radiotracer uptake in BAT during adrenergic stimulation is often used as a surrogate measure for identifying thermogenic activity in the tissue. BAT thermogenesis is believed to be contingent upon the expression of the protein UCP1, but conflicting results have been reported in the literature concerning [^18^F]FDG uptake within BAT of mice with and without UCP1. Differences in animal handling techniques such as feeding status, type of anesthetic, type of BAT stimulation, and estrogen levels were identified as possible confounding variables for [^18^F]FDG uptake. In this study, we aimed to assess differences in BAT [^18^F]FDG uptake between wild-type and UCP1-knockout mice using a protocol that minimizes possible variations in BAT stimulation caused by different stress responses to mouse handling.

**Results:**

[^18^F]FDG PET/CT scans were run on mice that were anesthetized with pentobarbital after stimulation of non-shivering thermogenesis by norepinephrine. While in wild-type mice [^18^F]FDG uptake in BAT increased significantly with norepinephrine stimulation of BAT, there was no consistent change in [^18^F]FDG uptake in BAT of mice lacking UCP1.

**Conclusions:**

[^18^F]FDG uptake within adrenergically stimulated BAT of wild-type and UCP1-knockout mice can significantly vary such that an [^18^F]FDG uptake threshold cannot be used to differentiate wild-type from UCP1-knockout mice. However, while an increase in BAT [^18^F]FDG uptake during adrenergic stimulation is consistently observed in wild-type mice, in UCP1-knockout mice [^18^F]FDG uptake in BAT seems to be independent of β_3_-adrenergic stimulation of non-shivering thermogenesis.

## Background

Brown adipose tissue (BAT) is a fat tissue found in most mammals, and its primary function is non-shivering thermogenesis (NST)—a homeostatic response to a cold stress, during which heat is produced without muscle contractions in order to maintain core body temperature [1]. Impaired BAT thermogenesis has been linked to metabolic diseases such as obesity and diabetes not only in rodents [2, 3], but also in humans, where the amount of detected BAT activity has been observed to be inversely correlated to body-mass index [4, 5]. Accordingly, in an effort to combat obesity, therapeutic approaches are being developed to specifically target BAT activation and or expansion [6]. In order to evaluate the efficacy of these treatments, imaging modalities that are able to accurately identify and quantify BAT thermogenesis are essential [7, 8].

[^18^F]FDG positron emission tomography/computed tomography (PET/CT) is the imaging technique most commonly used to assess BAT activity in rodents and humans [4, 7, 8]. Often, in these scans, an absolute threshold for the standardized uptake value (SUV), a measure of radiotracer accumulation, is used to identify and quantify thermogenically active BAT in the supraclavicular fat depots of humans [7–9]. [^18^F]FDG is also often used for cancer detection. But, due to the fact that SUV has a large degree of physical and biological variability, the practice of using SUV thresholds for cancer diagnosis is not widely accepted [10]. With this in mind, the use of an SUV threshold for validation of BAT thermogenesis should be met with similar apprehension.

Several articles have extensively reported on the many limitations of [^18^F]FDG/PET for the detection of BAT activity [7, 8], even suggesting that adrenergically induced glucose uptake in brown adipose tissue is independent of BAT thermogenesis [11]. These results seem to indicate that the high variability seen in human BAT glucose uptake [12, 13] may not be due to true differences in BAT thermogenesis [14], but to differences in insulin sensitivity and tissue blood flow [15].

Thermogenic activity in BAT is driven by the uncoupling protein 1 (UCP1), a protein that uncouples oxidative phosphorylation from adenosine triphosphate production in the mitochondria of brown adipocytes, resulting in increased heat production. It is widely accepted that BAT thermogenesis is contingent on the presence of functional UCP1 [16]. Therefore, wild-type (WT) and genetically modified knockout mice lacking UCP1 (KO) have been used to validate the use of [^18^F]FDG PET for the detection of BAT thermogenic activity. However, the current literature is comprised of conflicting results. In a study of only male mice, adrenergic stimulation led to an increase in [^18^F]FDG uptake in BAT of WT mice and not in KO mice, suggesting that [^18^F]FDG uptake is indeed a good measure of UCP1-mediated thermogenesis [17]. This finding was later supported by Jeanguillaume et al. who, this time, included both male and female mice. Unexpectedly, these authors observed [^18^F]FDG uptake in female KO mice, suggesting an effect due to sex [18]. This discrepancy between male and female KO mice was corroborated by Hankir et al., who suggested that uptake is actually independent of the expression of UCP1 [19]. Olsen et al. identified the UCP1-independent mTOR pathway as an alternative cause for [^18^F]FDG uptake upon BAT stimulation, but they did not observe the difference in uptake between male and female KO mice [11].

These conflicting results demonstrate the controversy of relying on [^18^F]FDG uptake to indicate BAT thermogenesis. Incorrect identification of BAT thermogenesis by [^18^F]FDG could be caused by a variety of confounding variables. Mouse handling is known to induce a variable stress-response in mice, possibly influencing [^18^F]FDG uptake in BAT. In addition, feeding status [1, 20], type of anesthetic [20–22], and method of BAT stimulation [23] are all possible confounding variables. Further, differences in estradiol levels and estrogen hormone concentrations, which depend on the current day in a female mouse’s estrous cycle, could explain the unexpected glucose uptake in female KO mice [24]. To this end, the aim of this study was to assess differences in BAT [^18^F]FDG uptake between WT and KO mice using a protocol that minimizes possible variations in BAT stimulation caused by a different stress response to mouse handling.

## Methods

### Animal handling protocol

All animal experiments were performed according to the ethical guidelines for animal experiments as described in the Public Health Service Policy on Humane Care and Use of Laboratory Animals [25], the Animal Welfare Act and Animal Welfare Regulations [26], and the Guide for the Care and Use of Laboratory Animals [27] under an animal protocol approved by the Institutional Animal Care and Use Committee of the University of North Carolina at Chapel Hill. For these studies, a colony of UCP1−/− (KO) and UCP1 +/+ (WT) was first established from a single breeding pair of heterozygous mice with the C57BL/6 genetic background, purchased from the Jackson Laboratory. Mouse genotypes were confirmed in 15-day-old mice by PCR of mouse tail DNA, performed offsite by Celplor LLC (Raleigh, NC), as well as by post-mortem immunohistochemistry staining of excised interscapular BAT. Throughout their lifespan, the mice were fed a regular chow diet, housed at a room temperature (24 °C), and exposed to a 12 h light/dark cycle.

PET/CT imaging studies were performed on two sets of mice: mixed male and female (Set 1) and all male (Set 2). Set 1 included 11 WT (6 female/5 male) and 22 KO (8 female/14 male) that were scanned unfasted. Set 2 included only male mice (9 WT and 9 KO) and were scanned after 10–12 h of fasting. Mouse weight is reported in Additional file 1: AF 1.

In order to avoid possible differences in BAT stimulatory conditions from other external factors that are known to significantly affect BAT stimulation in a non-controllable way (outside room temperature, stress from handling), all mice from both Set 1 and Set 2 were anesthetized for the entire duration of the imaging experiment, such that response to the same acute adrenergic stimulatory condition could be evaluated [21]. A surgical plane of anesthesia was achieved with an intraperitoneal injection of 70 mg/kg of pentobarbital (*Nembutal*, Abbott Laboratories), one of the few anesthetics that is known to not inhibit thermogenesis in BAT [28].

To prevent excess loss of heat in the anesthetized mice, immediately after administration of anesthesia and during radiotracer uptake, body temperature was actively maintained. Set 1 mice were placed on a heated plate with a surface temperature of 36 °C. Set 2 mice were placed into a closed box with a controlled ambient temperature of 34 °C. A rectal temperature probe, inserted 2.5 cm inside the anus, was used to monitor core body temperature (Reflex Signal Conditioner, Neoptix, Canada) [29].

### PET/CT imaging protocol

Once sedated, tail vein catheters were placed for radiotracer injection to minimize injection errors, and thus minimize errors in calculating [^18^F]FDG SUV. After reaching a surgical level of anesthesia, mice were injected subcutaneously with 1 mg/kg of norepinephrine (NE). After 10 min, [^18^F]FDG was administered via tail vein catheter at a dose of 12.5 ± 1.3 MBq in 100 μL saline.

All mice were randomized and imaged on a small animal PET/CT system (SuperArgus model, Sedecal, Madrid Spain) within a 3-h period. CT images were acquired with X-ray peak energy of 70 keV, a current of 0.3 mA, and 360 projections. Images were reconstructed using a FeldKamp algorithm with a nominal resolution of 0.105 mm. For PET imaging, 20 min of static acquisition was conducted in all mice at 45 min post-injection of [^18^F]FDG. Images were reconstructed with a 3D-OSEM algorithms and with a pixel size of 0.37 × 0.37 × 0.77 mm^3^. The PET resolution with the 3D-OSEM was 1.0 mm in the center of the FOV. SUV was calculated based on the animal body weight and injected dose.

Baseline data were collected from surviving mice of Set 2 one week after the initial experiment. In this case, the subcutaneous injection of NE was omitted from the imaging protocol.

### Infrared imaging protocol

Infrared temperature imaging measurements were taken on 6 WT and 5 KO surviving mice from Set 2. For these measurements, mice were again anesthetized with 70 mg/kg of pentobarbital and placed into a transparent plastic box maintained at a constant temperature of 34 °C. A small aperture on the lid of the plastic box was made to house the objective of a thermal imaging camera (FLIR E4, FLIR Systems, Inc.) to enable temperature imaging in the closed box. Rectal temperatures were continuously recorded, and once body temperature equilibrated to the surrounding temperature, mice were injected with 1 mg/kg of NE. Over the course of 40 min, infrared images were regularly captured with the thermal imaging camera placed at a distance of 0.2 m and with a thermal emissivity of 0.95.

### Immunohistochemistry analysis of BAT tissue

According to our previously published methods of immunohistochemistry analysis [30], mouse genotypes were also confirmed in excised BAT. For this analysis, interscapular BAT was surgically dissected from both WT and KO mice right after euthanasia and fixed overnight in 4% paraformaldehyde at 4 °C. Samples were then dehydrated, cleared, embedded in paraffin, and sectioned into 4 μm slices. Sections were then dewaxed and rehydrated. Cyto-Q Background Buster (NB306; In-novex) was used for the blocking procedure, followed by UCP1 primary antibody incubation at room temperature (1:1000; catalogue no. ab10983; Abcam). Secondary antibody incubation was performed with biotinylated goat anti-rabbit (1:1000, BA100, Vector) and detection performed with Vectastain Elite ABC complex (Vector). Slides were viewed on Aperio ImageScope (Version 12.3.3, Leica Biosystems Imaging, Inc.).

### Image analysis

[^18^F]FDG PET/CT images were analyzed using Horos (Nimble Co LLC d/b/a Purview, Annapolis, MD USA) imaging software by two investigators blinded to the animal genotype. After co-registration and fusion of corresponding PET and CT images, anatomical landmarks were used to identify the interscapular BAT depot posterior to the center of the upper thoracic vertebrae. Within this tissue, a circular region of interest (ROI) of constant area (5 mm^2^) was centered around the local maximum and SUV_peak_ was measured. SUV_peak_ represents the average uptake within an ROI centered around the local maximum, and it is less susceptible to image noise [31]. For Set 1, the WT and KO groups were further sorted and compared between male and female.

IR images were analyzed with FLIR Tools (Version 5.13.18031.2002, FLIR Systems, Inc.). ROIs were drawn in surface regions above BAT and non-BAT on both the WT mouse and the KO mouse before NE injection, and the corresponding maximum temperatures were recorded. Matching ROIs were drawn on images taken 40 min after NE injection, when the change in temperature was greatest in both mice, and the change in surface temperature in each region for each mouse was compared.

### Statistical analysis

Statistical analysis of the data was done by using the JMP Pro (Version 14, SAS Institute Inc., Cary, NC) software. All means are presented with their corresponding standard deviation. To compare [^18^F]FDG uptake, significant differences with *p* < 0.05 between groups were determined using two-way ANOVA for Set 1 and one-way ANOVA for Set 2, along with Tukey–Kramer HSD for multiple comparisons. Matched-pairs t tests were also used to evaluate the effect of NE on glucose uptake in BAT of WT and KO mice. Bartlett’s test was used to determine a difference in variance in SUV_peak_ with and without NE injection. Data were screened for outliers using the built-in JMP outlier analyses.

## Results

### Immunohistochemical and thermometry findings

**Fig. 1:**
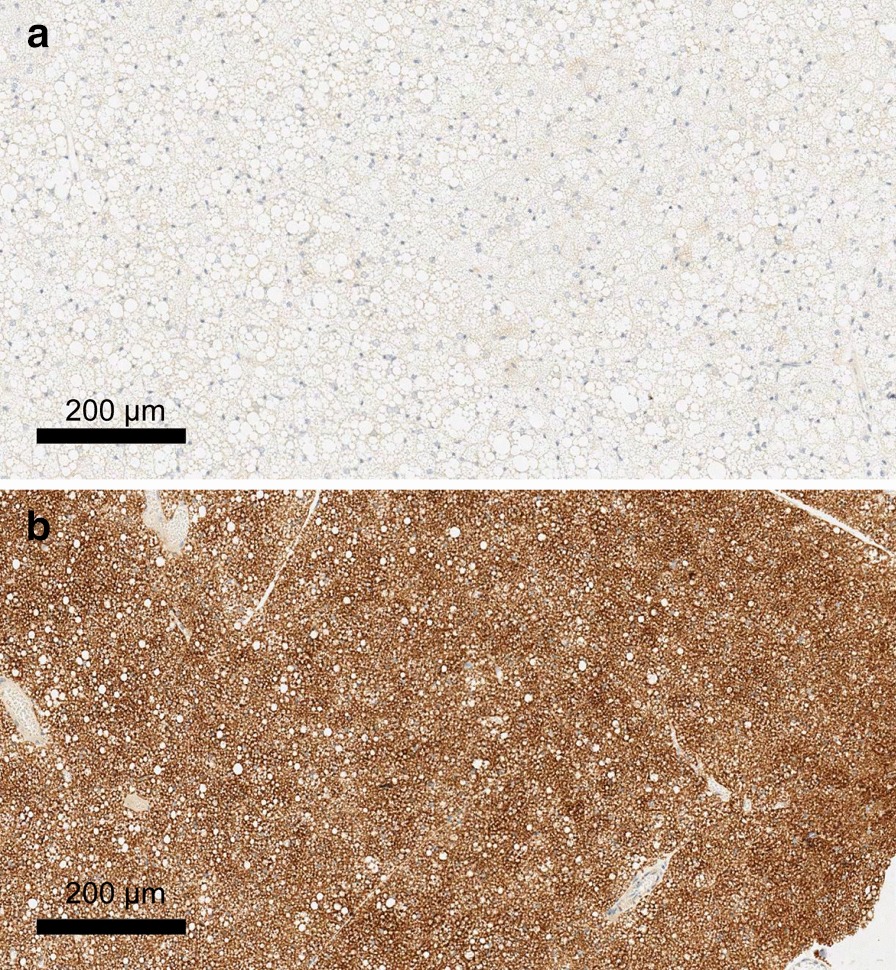
Immunohistochemistry staining of interscapular BAT dissected from WT and KO mice. **a** KO, **b** WT genotypes within breeding colonies were validated with UCP1 staining of dissected interscapular BAT. Slides are shown at 50% zoom. The lack of protein in our KO mice was confirmed, and UCP1 was strongly expressed in the BAT cells of our WT mice

The most physiologically relevant parameter that correlates with BAT thermogenic capacity is UCP1 protein expression level in BAT [32]. To this end, UCP1 staining by immunohistochemistry analysis was performed on excised interscapular BAT of both WT and KO mice. The results confirmed the complete absence of UCP1 protein in our KO mice, and strong expression of the protein in the brown adipocytes of our WT mice (Fig. 1). No visual differences in interscapular BAT mass or UCP1 protein expression level could be detected across the different WT mice.

**Fig. 2:**
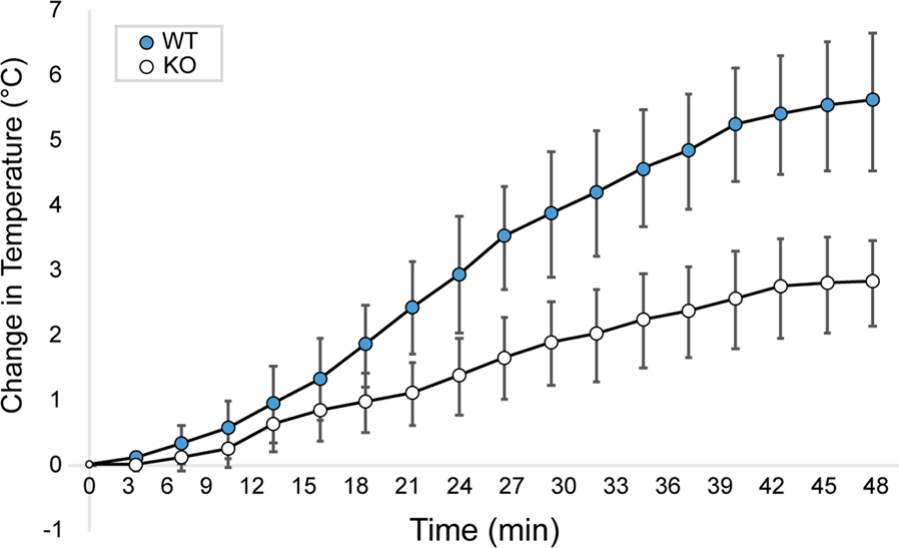
Average rectal temperature of male WT and KO mice after NE injection during [^18^F]FDG uptake for Set 2. To further corroborate the difference in BAT thermogenic capacity of our WT (blue) and KO (white) mice, rectal temperature was also recorded during the [^18^F]FDG uptake in the second set of experiments. Here, by studying mice of similar size, and by carefully controlling both NE dose and room temperature conditions, we were able to detect a significant (*p* < 0.001) difference in the increase in rectal temperature between WT and KO mice. Error bars represent the standard deviation at each time point

Rectal temperature measurements were taken on Set 2 to detect increases in core body temperature after adrenergic stimulation. Figure 2 shows the average change in rectal temperature of WT and KO mice immediately after NE injection. The increase in rectal temperature measured at 40 min after NE injection was markedly higher in WT mice (5.6 °C) than KO mice (2.8 °C) (*p* < 0.001). This difference should not be surprising, given the difference between UCP1 protein content in the interscapular BAT of WT and KO mice (Fig. 1). The observed increase in rectal temperature in KO mice after NE injection remains interesting, although not surprising. An increase in body temperature for KO mice may be due to UCP1-independent NE-induced metabolism [33]. Also, NE causes smooth muscle contraction and vasoconstriction, which is expected to lead on itself to a decrease in heat loss and to a consequential increase in core body temperature in both WT and KO animals. Nonetheless, the additional increase in heat production observed in WT mice is likely the result of UCP1-dependent thermogenesis.

Infrared thermometry measurements were taken on Set 2. Analysis of thermal images showed that, although skin temperature increased in all mice after NE injection, the measured temperature increase above the supraclavicular BAT region was not statistically different between WT and KO mice (*p* = 0.3). It is important to point out that these measurements were taken without shaving the interscapular area. Shaving of the interscapular area of anesthetized WT mice prior to infrared thermography was previously tried in our lab, and we observed that hair removal dramatically decreased insulation and increased heat loss, completely masking the surface body temperature increase due to BAT thermogenesis. For the current study presented in this paper, while infrared thermometry was able to detect body temperature increase in both animals following adrenergic stimulation, it was not able to detect differences in BAT thermogenic activity between WT and KO mice, which is consistent with previous findings [34]. Because the infrared thermometry data were inconclusive, data are not shown here but example images (AF 2a) and temperature data (AF 2b) are provided in the additional files.

### *[*^*18*^*F]FDG uptake in BAT of unfasted mice: Set 1*

**Fig. 3:**
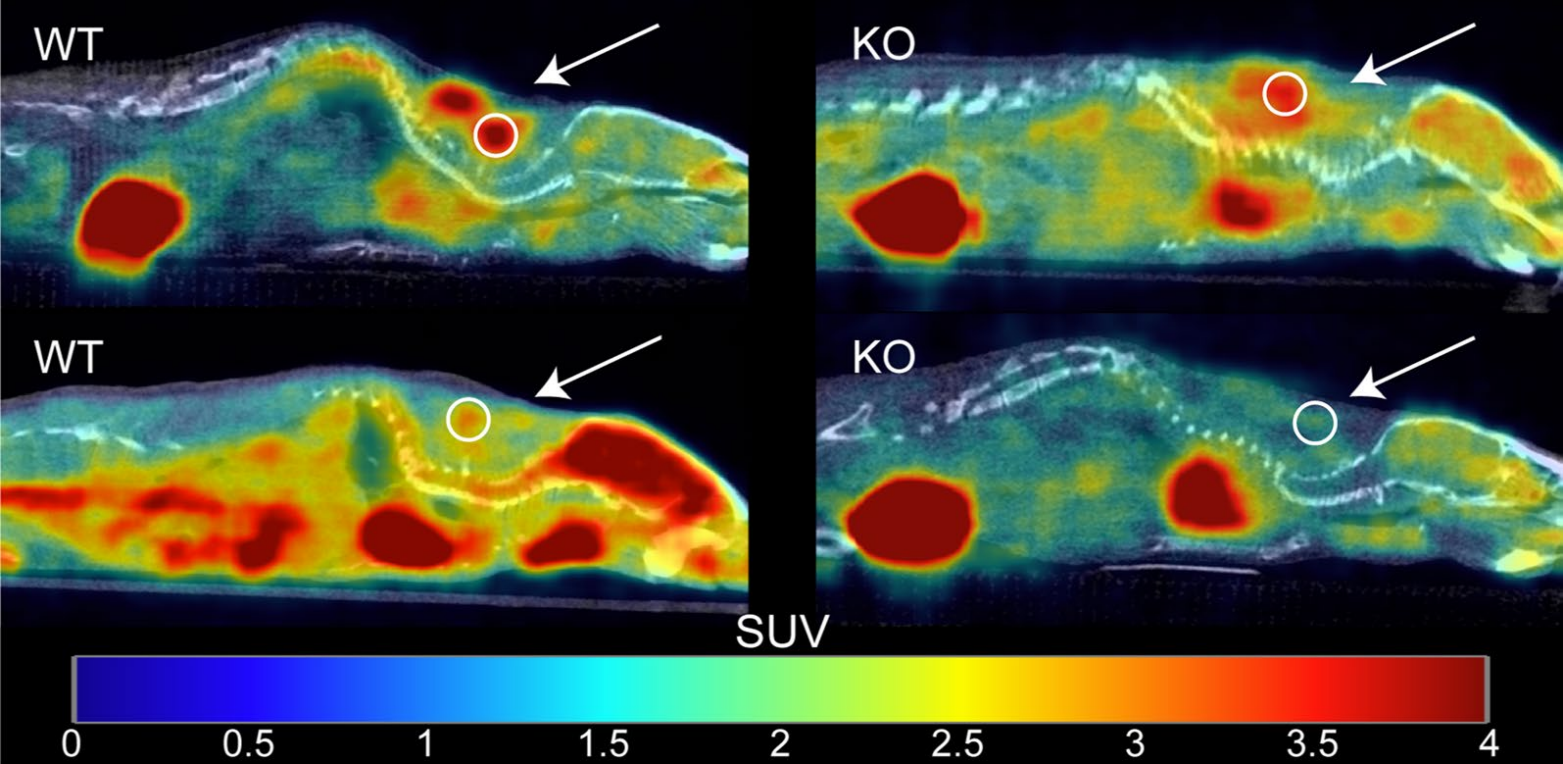
Example of fused [^18^F]FDG PET/CT images acquired from unfasted male WT and KO mice after NE injection. Images are displayed on the same SUV scale on a sagittal view. For each mouse, a 5 mm^2^ ROI (circle) was drawn around the region of maximum intensity within interscapular BAT (arrow), and the corresponding SUV_peak_ was calculated. Displayed on the left column are two different WT mice that present very different glucose uptake. Represented on the right column are two KO mice with very different [^18^F]FDG uptake

**Fig. 4:**
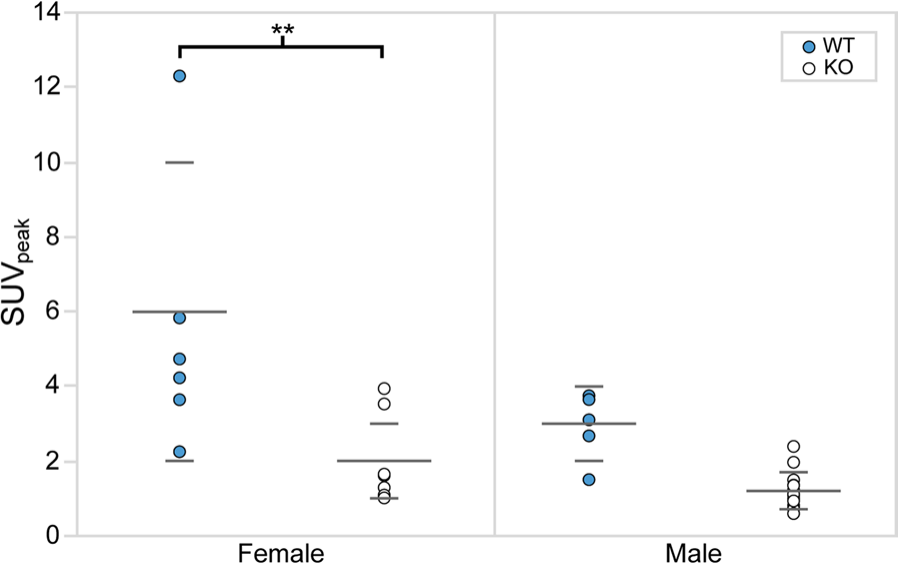
[^18^F]FDG SUV_peak_ within interscapular BAT of unfasted female and male mice after NE injection. SUV_peak_ is plotted for each genotype. Means (short) and standard deviations (long) for each group are represented by horizontal gray bars. A statistically significant difference between WT (blue) and KO (white) SUV_peak_ was only observed for female mice (*p* = 0.002). There was no evidence that the effect of the genotype depended on the sex of the mouse (*p* = 0.1)

Figure 3 shows examples of fused [^18^F]FDG PET/CT sagittal images acquired from male WT and KO mice. For each mouse, SUV_peak_ was measured in regions of interscapular BAT, and data between groups were compared using two-way ANOVA (Fig. 4). The data were screened for outliers, and it was determined that no data should be excluded. The mean SUV_peak_ for WT (mean = 4 ± 3) and KO (mean = 1.5 ± 0.9) groups were found to be significantly different (*p* = 0.0002). After further separation of the two groups by sex, a Tukey–Kramer HSD analysis showed that the difference between WT (female mean = 6 ± 4, male mean = 3 ± 1) and KO groups (female mean = 2 ± 1, male mean = 1.2 ± 0.5) was only seen between female WT and KO mice (*p* = 0.002), but not between WT and KO male mice (*p* = 0.2). In contrast with previous studies, there was no evidence that the effect of the genotype depended on the sex of the mouse (*p* = 0.1) [18, 19]. Interestingly, the data acquired on the first group of mice showed high variability within each subgroup. Specifically, we could not identify an absolute SUV_peak_ threshold that could be used to differentiate WT from KO mice due to the significant overlap of SUV_peak_ values between the two groups.

### *[*^*18*^*F]FDG uptake in BAT of fasted mice: Set 2*

**Fig. 5:**
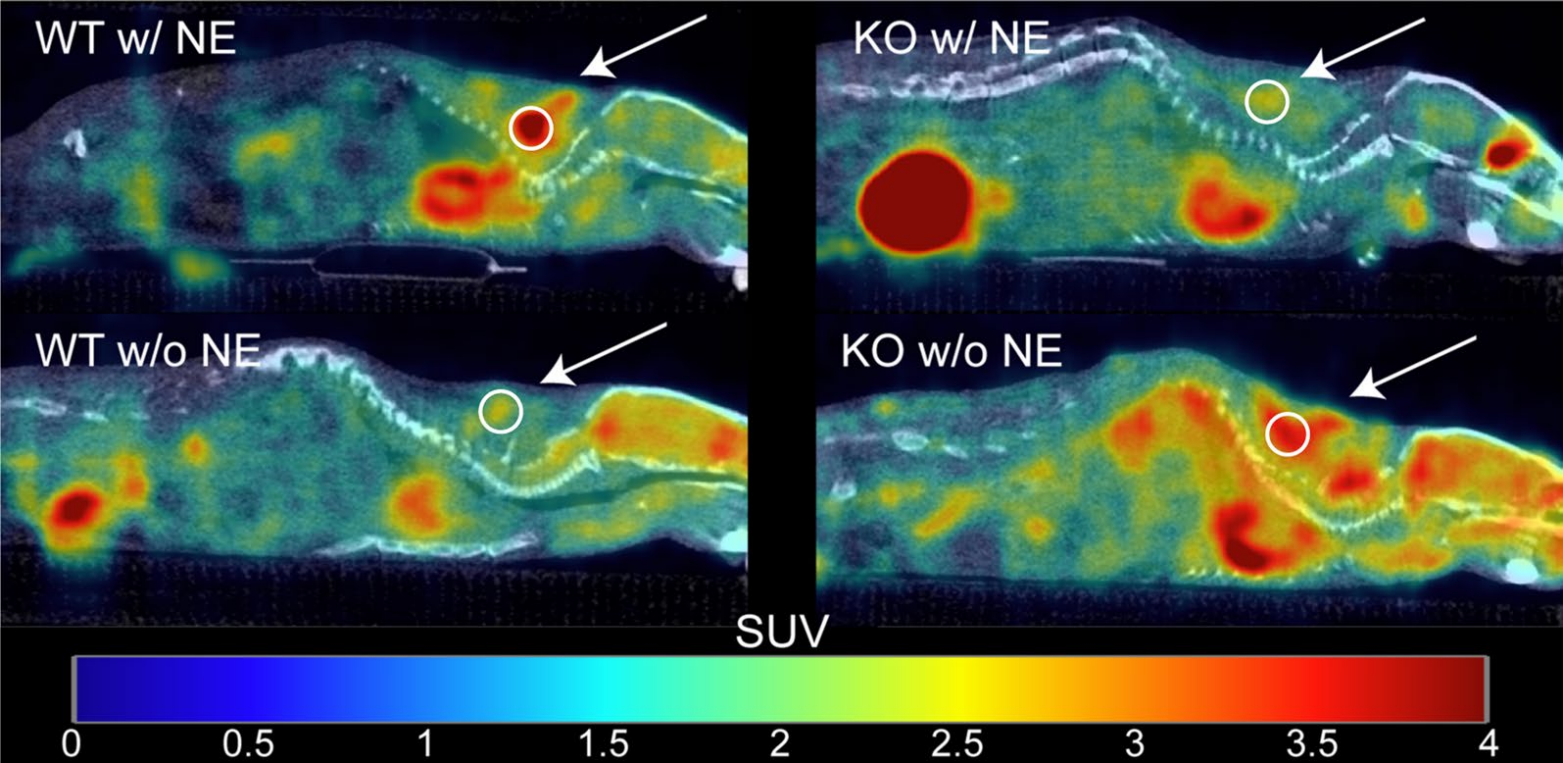
Example of fused [^18^F]FDG PET/CT images acquired from fasted male WT and KO mice with and without NE injection. Images are displayed on the same SUV scale on a sagittal view. Male mice from Set 2 were subject to scans with (top row) and without (bottom row) NE injection. The arrows indicate regions of interscapular BAT, and circles indicate the 5 mm^2^ ROI centered around the local maximum that was used to measure SUV_peak_. While uptake increases after NE injection for the WT mouse (left column), uptake for the KO mouse (right column) seems suppressed upon NE injection

**Fig. 6:**
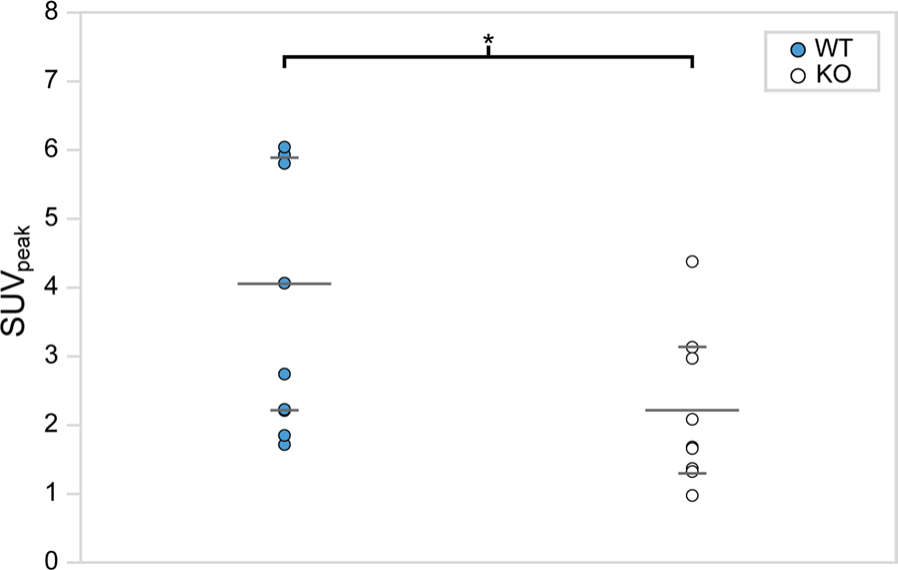
[^18^F]FDG SUV_peak_ within interscapular BAT of fasted male mice after NE injection. SUV_peak_ is plotted for each genotype. Means (short) and standard deviations (long) for each group are represented by horizontal gray bars. With fasting, a statistically significant difference between male WT (blue) and KO (white) SUV_peak_ was observed (*p* = 0.03). However, the WT and KO SUV_peak_ ranges still overlapped and a clear SUV threshold to differentiate animals with very different thermogenic capacity cannot be established, despite animal fasting

**Fig. 7:**
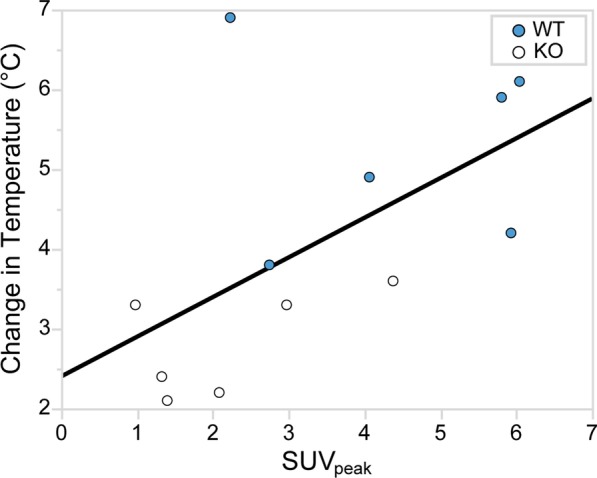
Correlation between rectal temperature and SUV_peak_. The change in rectal temperature during [^18^F]FDG PET/CT image acquisition is plotted against SUV_peak_ for Set 2. Although the data show a significant correlation (*p* = 0.046) between the change in rectal temperature and SUV_peak_ during NE stimulation of BAT thermogenesis, the increase in rectal temperature, in general, cannot be taken as a surrogate measure of BAT thermogenesis

Figure 5 shows examples of fused [^18^F]FDG PET/CT sagittal images acquired from male WT and KO mice. For each mouse in Set 2, SUV_peak_ was measured in regions of interscapular BAT, and data between genotypes were compared using one-way ANOVA (Fig. 6). The data were screened for outliers, and it was determined that no data should be excluded. The mean SUV_peak_ for WT (mean = 4 ± 2) was significantly higher than the mean SUV_peak_ for KO (mean = 2 ± 1) (*p* = 0.03). Interestingly, when average [^18^F]FDG uptake in non-fasted mice (Set 1) was compared to average [^18^F]FDG uptake in fasted mice (Set 2), we observed no differences for neither male WT (*p* = 0.9) nor male KO (*p* = 0.3). A direct comparison between SUV_peak_ and the change in rectal temperature after NE injection is shown in Fig. 7 and was found to be statistically significant (*p* = 0.046).

**Fig. 8:**
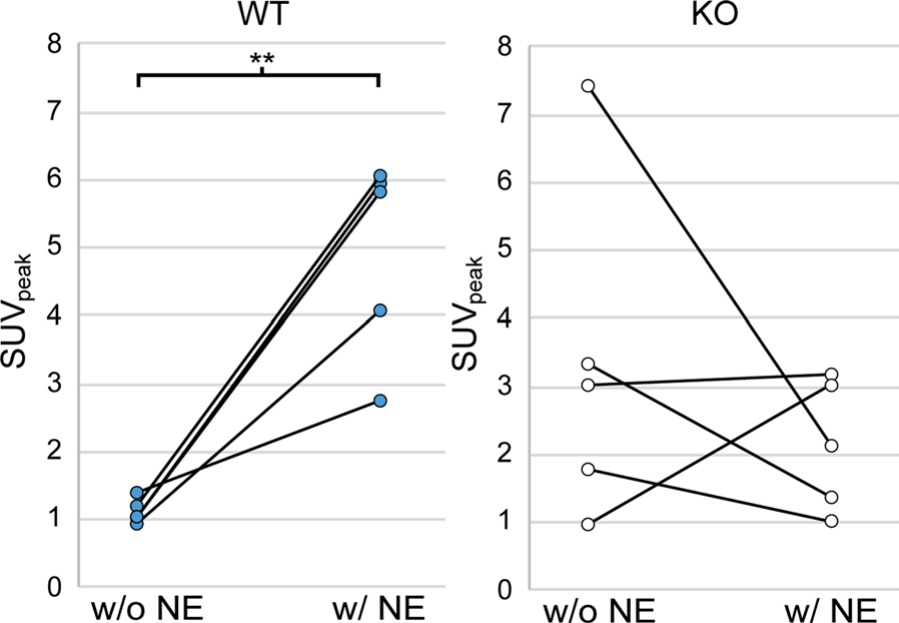
Effect of NE injection on SUV_peak_ for fasted male mice. SUV_peak_ is plotted for each genotype with (w/NE) and without (w/o NE) NE injection. There is a significant increase in the SUV_peak_ in the WT mice with NE injection (*p* < 0.001). On the other hand, SUV_peak_ in KO mice seems to either stay the same or decrease with NE injection. In addition, the average SUV_peak_ in KO mice without NE stimulation is much higher than that in WT mice (*p* = 0.046)

Ten surviving mice from Set 2 (5 WT and 5 KO) were subjected to a second [^18^F]FDG PET/CT scan a week later during which NE was not administered (baseline scan, Fig. 5). SUV_peak_ from the baseline data was compared to the stimulated data using a matched-pairs t test. At baseline, a significantly greater [^18^F]FDG uptake was observed in BAT of KO mice (x̄ = 3 ± 3) compared to WT mice (x̄ = 1.1 ± 0.2) (*p* = 0.046). Upon BAT activation with NE, a significantly greater increase in SUV_peak_ was observed in WT mice compared to KO mice (*p* = 0.01) (Fig. 8). In fact, there was no change in SUV_peak_ in the KO group (*p* = 0.4) between baseline and NE stimulated groups, while the increase in SUV_peak_ in the WT group was significant (*p* = 0.003). Interestingly, at baseline, the variance in SUV_peak_ of KO mice was higher than that of WT mice; the Bartlett’s test determined that the variances were significantly different (*p* < 0.001). Conversely, when the mice received the NE treatment, the variance in SUV_peak_ in the WT group increased, and the difference in variance in SUV_peak_ between WT and KO mice was no longer observed (*p* = 0.4).

## Discussion

BAT is a fat tissue that regulates energy balance and maintains core body temperature through sympathetic NST [1, 35]. Insufficient BAT activity could lead to an energy imbalance, resulting in metabolic diseases like obesity and diabetes. As such, BAT is a promising target for therapies and treatments against obesity [6]. Detection and quantification of BAT thermogenesis in humans are commonly done by using [^18^F]FDG PET/CT imaging after mild cold exposure [23]. However, to assess differences in BAT thermogenic activity across subjects, or to monitor changes in BAT thermogenesis in the same subject, [^18^F]FDG uptake ought to reliably reflect the degree of thermogenic activity in BAT. The aim of this study was to assess whether there exist differences in [^18^F]FDG uptake between animals with very different functional BAT thermogenic capacity, while addressing some of the inconsistencies found in the literature regarding varying [^18^F]FDG uptake patterns for mice with functional or impaired BAT [16–19].

Since animal handling in awake mice is known to lead to a considerable and variable stress response, resulting in the release of hormones and glucocorticoids that may influence the degree of BAT activity, all experiments were done in anesthetized mice. For these studies, all mice were anesthetized with pentobarbital, one of the few anesthetics that do not adversely affect BAT thermogenic capacity [21, 22].

In response to cold stress, NE is secreted and attaches to β_3_-adrenergic receptors on brown adipocytes to initiate the signal pathway for BAT thermogenesis. This provides UCP1-carrying mice the means to survive cold environments. But, UCP1-lacking mice are also capable of adapting to colder conditions by developing endurance for muscular shivering. Therefore, the participation of BAT in response to cold stress is completely optional [1]. In fact, since all mice were kept in a chronic mild cold environment at 24 °C, this could influence the baseline values observed for WT and KO mice. In order to isolate the effect of BAT thermogenesis on glucose uptake in BAT, we decided to inject NE directly into the anesthetized mice, bypassing any uncontrollable adaptive response to cold exposure that KO mice might have developed.

In preparation for [^18^F]FDG PET scans, it is common practice to have subjects fast. This is because elevated blood glucose levels have variable effect on SUV measured within different organs. For example, SUV in the brain and liver may decrease due to the endogenous glucose competitively inhibiting the uptake of the exogenous, glucose-analog radiotracer [36]. On the other hand, a previous study had shown a remarkable decrease in BAT uptake in non-anesthetized mice upon fasting [37]. For our study, experiments were run on two different sets of mice. Set 1 consisted of mixed-sex WT and KO mice that were scanned unfasted. Set 2 consisted of all male WT and KO mice that were scanned fasted. Interestingly, we did not see any significant difference in [^18^F]FDG uptake between fasted and unfasted mice (*p* = 0.3 for KO, and *p* = 0.9 for WT). This should not be surprising, though, as the reduction in glucose uptake seen in awake, fasted mice [37] is more likely due to the reduction in the sympathetic nervous system response of brown adipose tissue [38], which is clearly not seen in our studies in which BAT was directly stimulated by NE. Interestingly and somewhat counter-intuitively, fasting led to an even higher standard deviation of glucose uptake in BAT in both WT and KO mice. More insight could be gained in a future study by measuring individual glucose tolerance in order to normalize glucose uptake.

Figure 8 shows how NE injection affects SUV_peak_. For WT mice, SUV_peak_ increased significantly with NE stimulation of BAT, whereas there was no consistent change in [^18^F]FDG uptake in KO mice. At baseline, higher [^18^F]FDG uptake was seen in KO mice compared to WT mice. It is important to acknowledge the fact that our baseline scans were collected one week after the NE treatment scans. It has been shown that thermogenic activation can affect the functionality of BAT of KO mice through inflammation or other pathways [39, 40]. Our study would have benefitted from collecting baseline data before the treatment data. Thus, it is difficult to draw conclusions based on the comparison of means.

On the other hand, a comparison of the variances is much more interesting. At baseline, the variance for KO mice was much greater than WT mice (*p* < 0.001). But, with the inclusion of the NE treatment, the SUV_peak_ variances between KO and WT were comparable (*p* = 0.9). We hypothesize that this variability seems to suggest that, for WT mice, there is some UCP1-dependent mechanism that inhibits glucose uptake in absence of adrenergic stimulation, while facilitating glucose uptake upon stimulation. Glucose transport across cell membranes is facilitated by proteins such as glucose transporter 1 (GLUT1). Inokuma et al. [17] reported that GLUT1 mRNA levels were 1.4 times higher in KO mice when compared to WT mice. Therefore, even without stimulation, KO mice are expected to have higher [^18^F]FDG uptake due to higher expression of GLUT1. The functional activity of GLUT1 is enhanced after NE injection, and this may be due to a conformational change in GLUT1 that increases its affinity for glucose [41]. But, as shown in Fig. 5, this conformational change might be UCP1 dependent. In the presence of UCP1, GLUT1 might behave like a NE-gated channel that either restricts glucose uptake at baseline, or enhances glucose uptake upon NE stimulation. Conversely, for KO mice that lack UCP1, such control on GLUT1 might have lost, resulting in consistent changes and high variations in both baseline and stimulated conditions.

For Set 1, a wider range of SUV_peak_ was observed in female compared to male mice. In addition, a significant overlap of SUV_peak_ was seen between WT and KO female mice (Fig. 2). These findings are somewhat consistent with those observed by Jeanguillaume et al. [18] and Hankir et al. [19]. The wider range in SUV_peak_ observed in female mice could be ascribed to differences in estrogen levels, possibly present as mice were not scanned at the same estrous cycle time point [24]. As the resources required to monitor blood estrogen level were not available at our facility, to limit possible variations in SUV_peak_ due to differences in estrogen level, female mice were excluded from Set 2 for the second study. Future studies should explore the effect of estrogen levels, that could be measured with various kits for serum measurement [42] or urine samples [43], on glucose [^18^F]FDG uptake.

Differences in BAT thermogenic capacity between WT and UCP1 KO mice are well established in the literature. In our study, the lack of UCP1 in the BAT of KO mice and the presence of UCP1 in BAT of WT mice was established by genotyping of mouse tail DNA via PCR, as well as by immunohistochemistry staining of excised BAT. Differences in BAT thermogenic capacity between the two phenotypes were also assessed by rectal temperature measurement and by using infrared thermography. Rectal temperature measurements showed a much higher core body temperature increase in WT mice than KO mice 40 min after NE injection (Fig. 2), further demonstrating different NE thermogenic responses between WT and KO mice. Further, Fig. 7 shows a positive correlation between the change in rectal temperature and SUV_peak_ (*p* = 0.046). Although this might suggest that direct rectal temperature measurements sufficiently indicate BAT thermogenesis, this is generally not the case. This is due to the fact that, from a thermodynamics viewpoint, rectal temperature (or internal body temperature) depends on the amount of heat produced by various metabolic processes (including the heat produced by BAT) and the amount of heat lost to the environment. The latter is modulated by animal size, fur (which, when removed, leads to a significant decrease in animal heat capacity and increase in heat loss, thus masking any possible increase in rectal temperature), vascular response (increase vasoconstriction and blood flow), and external room temperature, all of which would need to be carefully monitored and independently measured in order to extract quantitative data on BAT thermogenesis.

Most likely because of these reasons, infrared thermography did not show any statistically significant difference in thermogenic capacity between WT and KO mice, consistently with previous studies done in unconscious mice showing the inability of thermal imaging to show changes with CL-316,243 [44]. As such, in our study, while infrared thermometry was able to detect body temperature increase in both animals following adrenergic stimulation, it was not able to detect differences in BAT thermogenic activity between WT and KO mice, which is consistent with previous findings [34].

## Conclusions

While undoubtedly there exists a clear difference in BAT thermogenic activity between WT and KO mice based on their UCP1 expression and the increase in core body temperature caused by NE and measured by rectal temperature probes, differences in [^18^F]FDG uptake between WT and KO mice are not as indicative. Also, the mechanism by which [^18^F]FDG is shuttled into BAT is not completely understood in KO mice. The origin of the high variability in the magnitude of [^18^F]FDG uptake in these mice is unclear. These results, at a minimum, should cast doubt on the use of SUV as a surrogate measure for the quantification, but not for the detection, of BAT thermogenesis, at least in mice.

## Supplementary information

**Additional file 1.** Means (long bars) and standard deviations (small bars) for each group are represented by horizontal grey bars. For female mice, the average weight for WT and KO genotypes were 23±3 g and 19±4 g, respectively. For male mice, the average weight for WT and KO genotypes were 29±3 g and 28±6 g, respectively.

## Data Availability

The dataset supporting the conclusions of this article is available in the Carolina Digital Repository, https://cdr.lib.unc.edu/collections/05741x435?locale=en.

## Abbreviations

BAT: Brown adipose tissue
[^18^F]FDG: 2-[^18^F]fluoro-2-deoxy-d-glucose
KO: Knockout, lacking UCP1
NST: Non-shivering thermogenesis
NE: Norepinephrine
PET/CT: Positron emission tomography/computed tomography
ROI: Region of interest
SUV: Standardized uptake value
SUV_peak_: Peak standardized uptake value
UCP1: Uncoupling protein 1
WT: Wild type, expressing UCP1
