# Supplementary material for: Differences in [18F]FDG uptake in BAT of UCP1 −/− and UCP1 +/+ during adrenergic stimulation of non-shivering thermogenesis
Source: EJNMMI Res. 2020 Nov 7;10:136. doi: 10.1186/s13550-020-00726-x (PMC7648812; doi:10.1186/s13550-020-00726-x)
Supplement: Supplementary file 1 — Additional file 1. Means (long bars) and standard deviations (small bars) for each group are represented by horizontal grey bars. For female mice, the average weight for WT and KO genotypes were 23±3 g and 19±4 g, respectively. For male mice, the average weight for WT and KO genotypes were 29±3 g and 28±6 g, respectively. [file 13550_2020_726_MOESM1_ESM.docx]

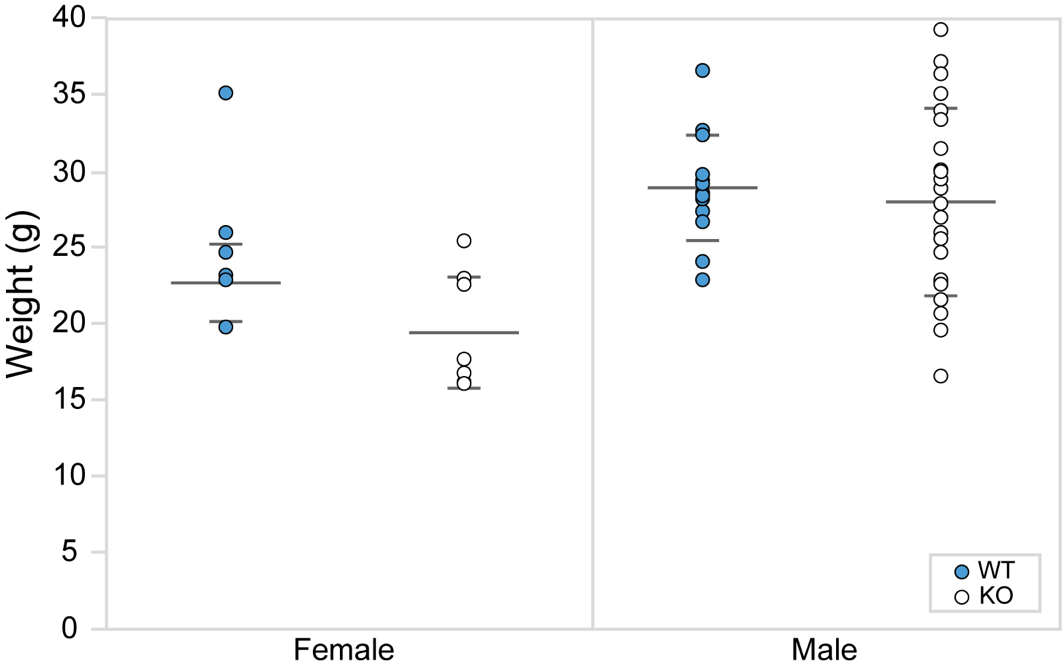


**AF1. Distribution of mouse weight across Set 1 and Set 2.** Means (short) and standard deviations (long) for each group are represented by horizontal grey bars. For female mice, the average weight for WT and KO genotypes were 23±3 g and 19±4 g, respectively. For male mice, the average weight for WT and KO genotypes were 29±3 g and 28±6 g, respectively.

**
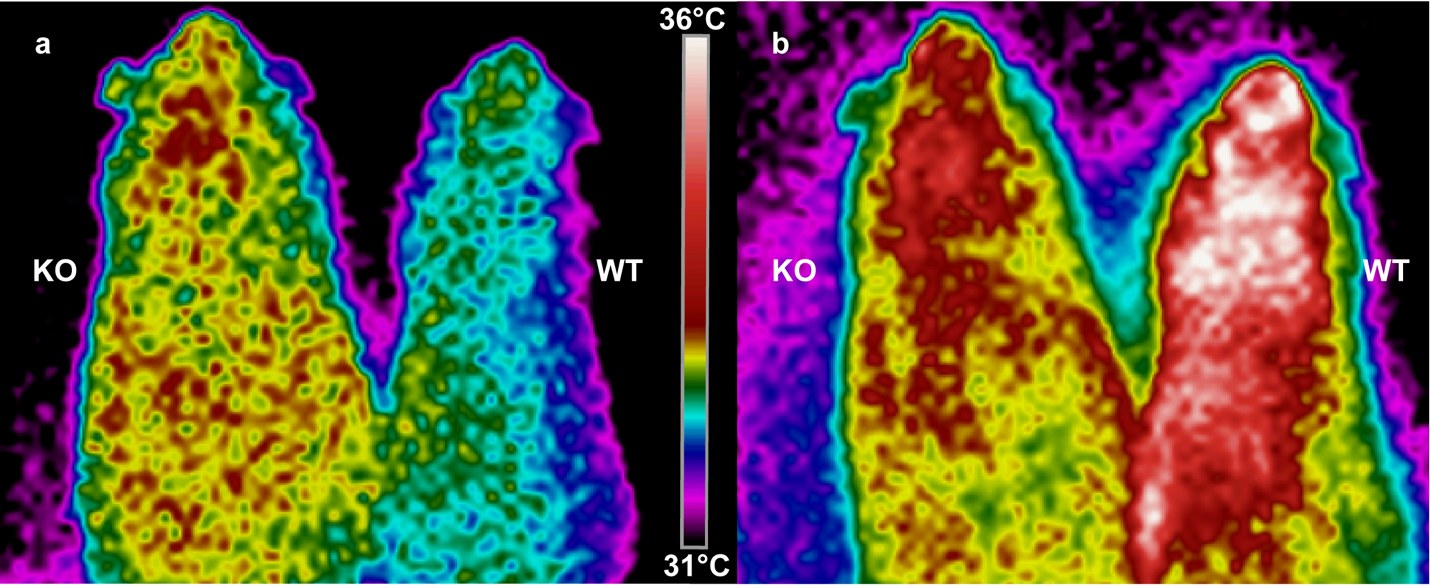
**

**AF2a. Representative thermal images of a male WT and a male KO mouse before (a) and after (b) BAT activation.** WT mouse is shown on the right of the two images, while KO mouse is shown on the left of the two images. Thermal images were taken over the course of 40 minutes while the animals were kept in an environment regulated to 34°C. Here we are showing images from baseline (a) and 40 minutes after injection (b). Regions above suprascapular BAT, as well as non-BAT areas, show an overall increase in body temperature in both mice after NE injection. However, no differences were measured in the magnitude of the increase in BAT temperature between WT and KO mice (p=0.3).

**
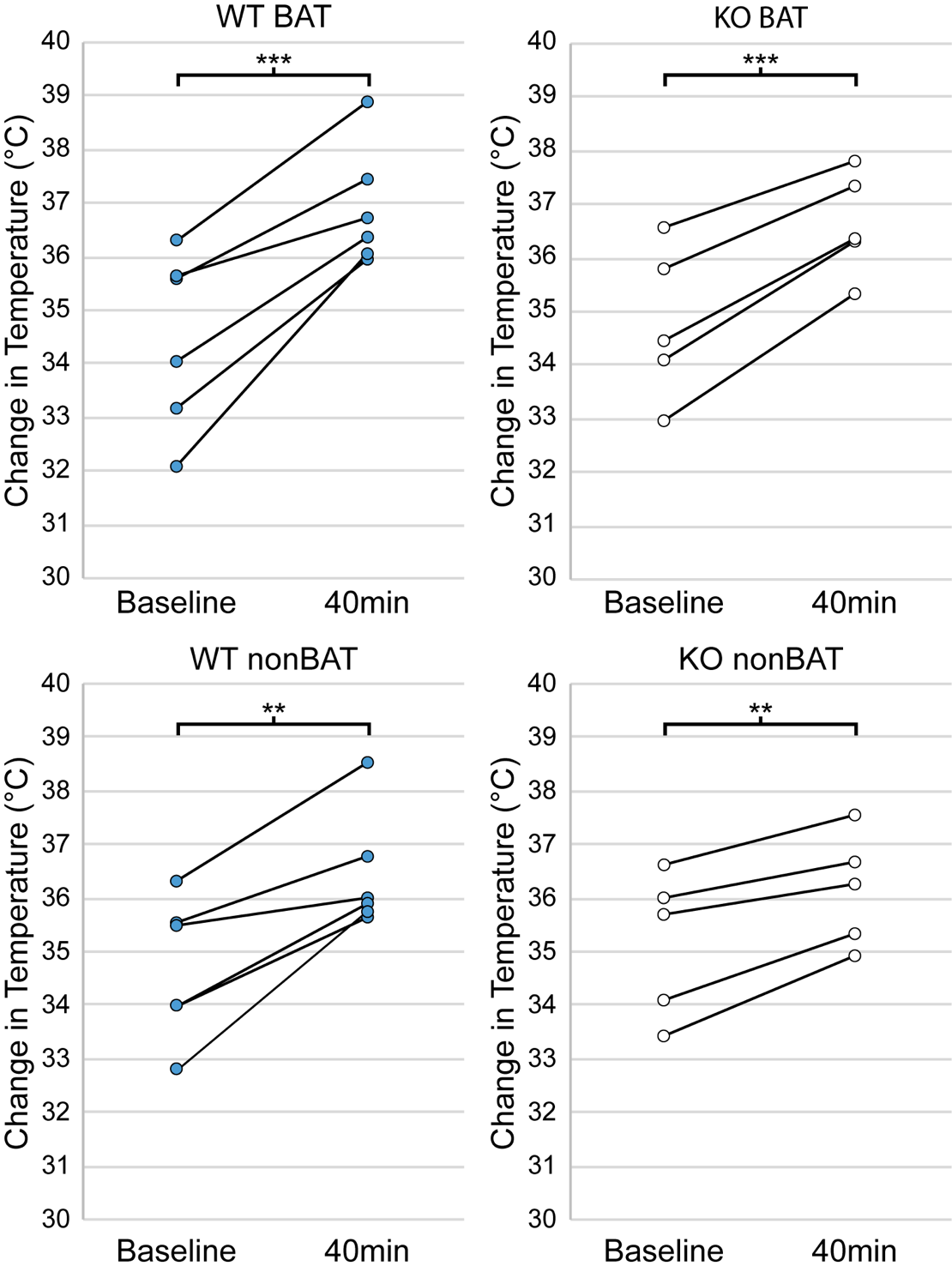
**

**AF 2b. Change in surface temperature 40 minutes after NE injection.** Plots showing the change in mouse surface temperature, as measured by infrared thermography, in regions above interscapular BAT (top row) and regions above non-BAT (bottom row). The increase in body temperature was statistically significant for both WT (left column) and KO (right column) mice in both regions. However, a matched-pairs t-test showed that there was no a significant difference in BAT temperature increase between WT and KO mice (p=0.3 above BAT and p=0.1 above non-BAT).
